# Short Sickness Absenteeism Rate in a Large Tertiary Hospital: The Role of Fitness for Work and Influenza Vaccination

**DOI:** 10.3390/epidemiologia7040096

**Published:** 2026-07-09

**Authors:** Alberto Lontano, Luca Inguaggiato, Daniele Ceriotti, Riccardo Rescinito, Enrico Oddone, Stefano M. Candura, Matteo Ratti

**Affiliations:** 1 AOU Maggiore della Carità di Novara—Tertiary University Hospital, 28100 Novara, Italy; alberto.lontano@uniupo.it (A.L.); 10033325@studenti.uniupo.it (R.R.); 2 National PhD Program in Artificial Intelligence, Healthcare and Life Science Area—XLI Cycle, Università Campus Bio-Medico di Roma, 00128 Roma, Italy; 3 Department of Science and Technological Innovation (DISIT), Università del Piemonte Orientale, 15121 Alessandria, Italy; 4 Department of Translational Medicine (DiMeT), Università del Piemonte Orientale, 28100 Novara, Italy; 20063254@studenti.uniupo.it; 5 Occupational Safety and Prevention Service (SPreSAL), ASL NO (Local Health Authority), 28100 Novara, Italy; danielecerio@gmail.com; 6 Occupational Medicine Unit, Department of Public Health, Experimental and Forensic Medicine, University of Pavia, 27100 Pavia, Italy; enrico.oddone@unipv.it (E.O.); stefano.candura@icsmaugeri.it (S.M.C.); 7 Istituti Clinici Scientifici Maugeri IRCCS, Occupational Medicine Unit of Pavia Institute, 27100 Pavia, Italy; 8 Doctoral Program in Food, Health, and Longevity, University of Eastern Piedmont, XXXIX Cycle, 28100 Novara, Italy

**Keywords:** sickness absenteeism, fitness for work, influenza vaccination

## Abstract

**Background:** Sickness absenteeism has long been an organizational problem due to the consequent loss of productivity (1–2% of gross domestic product in European Union countries) and job overload for workers. We designed and conducted an observational cohort study to describe and analyze the short sickness events and their derived absence days in a large university tertiary hospital, also incorporating the fit for work assessment and the anti-influenza vaccination coverage. **Materials and Methods:** The anonymous data of workers with at least one day of certified presence during the period 1 January–31 August 2025 were extracted from the hospital information system. Crude and job-specific absence rates were calculated. The incidence rate ratios (IRRs) along with their 99% confidence intervals for the covariates were obtained by a negative binomial model fit. **Results:** The overall sickness event rate was 0.54 per 100 person-workdays, ranging from 0.22 (physicians) to 0.92 (technical/administrative staff), while the overall sickness day rate was 0.88 per 100 person-workdays, spanning from 0.31 (physicians) to 1.41 (health and social care assistants). The multi-variable model fit of sickness days returned significant IRRs for being vaccinated (0.74; 99% CI: 0.58–0.93), for male gender (0.77; 99% CI: 0.63–0.95) and for limitations regarding the fitness for work assessment (2.14; 99% CI: 1.51–3.05). **Conclusions:** Our findings indicate that, in a large tertiary university hospital, an anti-influenza vaccination campaign may effectively protect workers from sickness absences, even accounting for gender, age and regardless of job typology and fit for work assessment. Furthermore, the health risk of the technical/administrative job category might be underestimated. Finally, our results seem to suggest that workers with limitations might be “fragile”, or more vulnerable, than expected in a broader sense of the term, both from nosological and temporal points of view. Further research with a higher level of evidence is needed to verify and confirm these findings.

## 1. Introduction

Sickness absenteeism, particularly in hospitals, has long been recognized as an organizational problem [1] due to the consequent loss of productivity and increased workload for the remaining staff. In fact, it is reported to be responsible for an estimated loss of 1–2% of the gross domestic product in the EU countries [2] and the source of a triple cost: financial, organizational, and cultural [3]. Recent estimates of the phenomenon show that the trend is increasing: a recent study of a large Italian hospital found that, from 2018 to 2023, the average absence days per employee rose by 2.86 days, with a sharp increase in leaves related to illness and injury (+4.6%) [4]. Also, the National Institute for Occupational Safety and Health (NIOSH) surveillance system in the United States reports a 3.01% overall rate of health-related workplace absenteeism for February 2025, compared to 2.54% for February 2024 and 2.43% for February 2023 [5]. Moreover, the rates appear to be heterogeneous with regard to productive sector and job typology: during 2016–2017, the UK National Health System (NHS) reported an absence rate of 4.2% for the aggregated staff, discriminating between doctors (1.1–1.2%), nurses (4.5%), and ambulance workers (5.5%). The main reasons for the leaves were minor illnesses such as cough and cold (33.7%) and musculoskeletal problems (18.1%) [6]. Notably, the healthcare sector reported an overall absence rate of 3.5%, which turned out to be higher than the 2% for all UK workers. It is also known that the disruptive effects of absenteeism could compound because absence may structure itself into a vicious cyclical relationship that leads to burnout or overload of workers, which in turn produces even more absence events [7].

The fitness for work assessment is a mandatory yearly medical examination of the worker. During this visit the doctor relates the risk that he/she faces when doing a specific job with his/her health status. The aim is to avoid tasks that may impair or worsen the health of the worker. The result of the process is a judgment that can be “fit for work”, “fit with prescriptions”, “fit with limitations”, “unfit”, or “fit with limitations due to pregnancy status”. Briefly, a prescription is a special equipment that the worker must wear when doing a specific task, whereas a limitation is a forbidden task. The pregnancy limitations are a specific set of standard limitations coded in a single law, but most of the law concerning workplace health is included in the national D.L.vo 81/2008. According to our preliminary literature research, the fitness for work assessment has never been considered in absenteeism analysis, but it may play a significant role when certain prescriptions or limitations are applied to the healthcare worker (HCW). Moreover, this assessment may also be seen as a concise judgment about the health condition of the worker, although it is restricted to job-related factors or risks and therefore may carry wider significance than what is usually recognized [8]. Concerning the association between sick leaves and respiratory infections, instead, we found that it has already been highlighted in previous research: the Observatory of the Italian National Providence Institute (INPS) [9] noted a marked seasonality of sickness leaves (with the second semester of the year registering decreases of about −20%), which closely reflects the circulation of respiratory viruses, as reported by the national surveillance system [10]. In fact, seasonal influenza still poses a public health problem, especially for elderly individuals and HCWs [11,12,13]: with an annual incidence of 40–50 million symptomatic infections in the entire European Union, this pathogen causes an estimated 21,000 excess hospitalizations and 16,000 avoidable deaths [14,15].

The World Health Organization identified HCWs as being at high risk of contracting an influenza-like illness (ILI) and therefore set a target of 75% for anti-influenza vaccination coverage in order to contrast the derived absenteeism and the possibility of transmission to vulnerable patients [16]. As a matter of fact, a recent systematic review has estimated a relative risk for vaccinated workers of contracting influenza of 0.36 (95% CI: 0.25–0.54), an ILI risk of 0.69 (95% CI: 0.45–1.06), an absenteeism rate of 0.63 (95% CI: 0.46–0.86) and a standardized mean difference of −0.18 (95% CI: −0.28 to −0.07) lost working days per person compared to unvaccinated colleagues [17], highlighting the importance of HCW vaccination in controlling epidemics in healthcare facilities. However, recent studies with mathematical models yielded conflicting results, stating that the best strategy in nursing homes might be to vaccinate guests rather than HCWs [18]. Another study showed a statistically significant difference among personnel who reported at least one sick day: it was found that vaccinated individuals experienced lower symptomatic days absent (OR 0.81; 95% CI: 0.69–0.95) [19].

The risk for HCWs of contracting a respiratory disease and, in turn, being absent seems to differ among job typologies: a recent systematic review with a meta-analysis indicated a higher risk for “frontline” workers who are directly involved in prompt care of patients (e.g., emergency department workers). In detail, they carried an aggregate OR of 1.66 (95% CI: 1.24–2.22) when compared to the other workers [20]. A retrospective study covering 13 seasons stratified the risk according to HCW category: when compared to unexposed workers (i.e., without inter-human contact), the risk of contracting influenza was higher for physicians (OR 3.21; 95% CI: 2.78–3.73), followed by medical and pathology laboratory technicians (OR 2.31; 95% CI: 1.69–3.15), nurses (OR 1.75; 95% CI: 1.55–1.97), and health assistants (OR 1.72; 95% CI: 1.57–1.87) [21]. However, we could not find any study investigating how job-specific exposure to biological agents translates into different rates of absenteeism and lost productivity. This could be relevant when designing occupational vaccination campaigns for HCWs so as to set the proper priority and achieve maximum effectiveness. Moreover, we retrieved no source of evidence investigating how fitness for work assessment may affect this relationship, and therefore we still do not know if this assessment may be used to effectively prioritize the campaigns.

For these reasons, we designed and conducted an observational cohort study in a large tertiary university hospital with the following objectives:

1. To describe the sickness events and related absenteeism of a cohort of hospital workers;
2. To analyze any difference in days of absence due to anti-influenza vaccination status, fitness for work assessment and job typology.

## 2. Materials and Methods

The study is described according to the Strengthening the Reporting of Observational Studies in Epidemiology (STROBE) methodology [22]. The relative compiled checklist is included in the Supplementary Materials (Table S1).

### 2.1. Study Aim and Design

The study was designed as an observational historical cohort study. The primary aim of the study was to describe and analyze the lost productivity due to sickness leaves of the workers of a tertiary university hospital, accounting for job typology, fitness for work assessment and vaccination against seasonal influenza.

### 2.2. Study Setting

The study was conducted at the University Teaching Hospital “Maggiore della Carità” in Novara, a tertiary referral center employing more than 3000 workers and with 540 beds. During 2025, the hospital managed 33,318 hospitalizations.

### 2.3. Study Population

The study population consisted of hospital workers and equivalent (e.g., physicians in residency training), employed in any position subject to health surveillance as required by the internal risk assessment document, which is mandatory by law. Although healthcare workers constituted the primary focus, all jobs subjected to health surveillance were included in order to provide a comparison with employees without exposure to biological hazards. For the eligible workers, the fit for work assessment is done once a year.

Inclusion criteria were:

- At least one day of certified presence at work in the following period: 1 January–31 August 2025;
- Presence on payroll register on 1 September 2024 so that the worker had the opportunity of participating in the influenza vaccination campaign.

The exclusion criteria were:

- Unfitness for work at the assessment visit;
- Job category that did not require health surveillance or “fitness for work” visit, as defined by the internal risk assessment document.

### 2.4. Study Outcomes

The main outcomes of the study were the number of sickness events and their related sickness absence days in the sample of workers during the period 1 January–31 August 2025. Even though the influenza virus and other respiratory infections are more frequent during the last days of the year, we wanted to isolate the vaccination effect and study a well-defined population at the cost of a probable underestimation of the crude annual rates (see Section Strength and Limitations). We only considered sickness events lasting a maximum of 5 days because they are more likely associated with infectious seasonal events [23,24]. Moreover, long-term absences are confirmed to be associated with chronic conditions [25].

The main outcome measure metrics were defined as follows:

1. Crude and job-specific rates of sickness events per 100 person-workdays;
2. Crude and job-specific rates of sickness days per 100 person-days;
3. Incidence rate ratios (IRRs) and 99% confidence intervals calculated by uni-variable and multi-variable generalized linear model fit, accounting for anti-influenza vaccination, gender, age, fitness for work assessment and job typology (see Section 2.5—Data Collection and Management).

When a holiday or weekend occurred between two sickness days, the sickness episode was not considered interrupted and counted as a single event. In fact, it is known that workers tend not to go to their general practitioners to be certified when they are on holiday or weekend, so the limitation to be present at home for a possible inspection (which would instead persist) does not apply even if the worker is still sick [26].

### 2.5. Data Collection and Management

The data was anonymously extracted from the hospital information system (therefore, no worker ID or other identifying parameters of the individual were extracted). Immediately after the extraction the queries were erased, and a k-anonymity analysis (k = 3) was conducted to ensure that no combination of the extracted variables might lead to the re-identification of the individual worker. The following variables were extracted:

- Age on 1 January 2025 (continuous variable—years).
- Gender (binary categorical variable—male and female).
- Influenza vaccination acceptance during the last seasonal in-hospital campaign, 1 September–31 December 2024 (binary categorical variable—yes and no).
- Job typology (categorical variable—nurse/physician or equivalent (e.g., resident doctor)/health and social care (HSC) assistant/technical or administrative staff/other healthcare worker (HCW)). The other HCW category includes physiotherapists, occupational therapists, midwives, and biomedical laboratory technicians.
- Result of the last fitness for work assessment visit (categorical variable—fit for work/fit with pregnancy status limitations/fit with prescriptions/fit with limitations/fit with both prescriptions and limitations/not fit for work). The fitness for work assessment is performed yearly by a dedicated ward of the hospital to any worker whose job is identified as involving a health risk by the internal risk assessment document.
- Number of sickness events that lasted 5 days maximum during the period 1 January–31 August 2025 (continuous variable).
- Number of sickness days of the events that lasted 5 days maximum during the period 1 January–31 August 2025 (continuous variable).

Even if the EU General Data Protection Regulation (GDPR—law 2016/679) does not apply to anonymous (non-personal) data, data management was conducted under its principles. Therefore, the dataset is kept in a password-protected computer with an encrypted filesystem (AES-XTS 128-bit).

### 2.6. Data Analysis

The dataset was kept in a csv plain text file format, stored in an encrypted file system. All the collected variables were described in tables and charts. Continuous variables were summarized by means and standard deviations or medians and interquartile ranges (IQRs) depending on the nature of their distributions. Categorical variables were described with counts and relative proportions. The description of sickness events was conducted by calculating crude and job-specific rates: for the sickness events, the denominator consisted of person-workdays since holidays and weekend days do not carry risk (or carry negligible risk) of being the first day of a sickness event; for the sickness days, instead, the denominator consisted of the total person-days of the considered period.

The incidence rate ratios (IRRs) were modeled by fitting a generalized linear model with Poisson or negative binomial regression (depending on whether over-dispersion was detected). As an offset of the regression model, working days were selected for sickness events and total days for sickness days. We calculated the 99% confidence intervals for the resulting model coefficients. The regression was conducted for the following variables: gender, age, anti-influenza vaccination acceptance, job typology, and fitness for work assessment. Only the covariates that turned out to be significant via the uni-variable analysis were included in the multi-variable models. The most frequent categories were employed as references.

Given that all the workers from the hospital were considered for inclusion in the study, no preliminary sample size calculation was conducted and the statistical significance (error of I type/alpha) was set at 0.01. Analyses were performed using R ver. 4.4.0 [27] and RStudio ver. 2023.09.1 Build 494 [28] with packages tidyverse [29] (ver.2.0.0), tableone [30] (ver.0.13.2) and MASS [31] (ver.7.3-60.2).

### 2.7. Potential Bias and Confounders

The literature about absenteeism provided evidence of an association with age and gender [32,33]; we therefore included these covariates in the multi-variable model fit regardless of the significance of the coefficients in the uni-variable analysis. Moreover, we restricted the study timespan to the first 8 months of the year because, by September, the new vaccination campaign would have started and the results could have been biased by the two overlapping campaigns. Since it is known that sickness absenteeism is disproportionately higher in the last period of the year, we expect our rates to be lower than the annual crude rates most studies have calculated.

### 2.8. Ethical Considerations

The study was conducted according to the Declaration of Helsinki. The authorization of the medical directorate of the hospital was obtained, and the institutional Ethics Committee approved the study with protocol number 087/CE of 29 January 2026 (application no. E00081/2025).

## 3. Results

### 3.1. Sample Description

Our sample consisted of 3218 workers: 1073 nurses (33.3%), 976 physicians or equivalent staff (30.3%), 454 HSC assistants (14.1%), 358 other HCWs (11.1%), and 357 technical/administrative workers (11.1%). Most of our sample was composed of women (72.0%), ranging from 58.5% of physicians to 83.7% of HSC assistants across job typologies. The mean age appeared to be slightly higher (circa 54 years) in the technical/administrative category, while the physicians were the youngest group, with a mean age of 41.84 years. Overall, 21.3% of the sample had an anti-influenza vaccination, with substantial variability: the HSC assistants recorded the lowest acceptance (only 11.2%), while the physicians were the most prone to receive the vaccination (35.0%). Two thirds of the workers (66.2%) were judged “fit for work”, with the highest proportion (78.3%) among physicians, followed by technical/administrative staff (72.0%), other HCWs (63.4%), and nurses (61.0%). The HSC assistant category displayed the lowest proportion of “fit for work” individuals (50.2%) but the highest in all of the other modalities except the limitations due to pregnancy status (fit with limitations: 11.5%; fit with prescriptions: 26.7%; fit with both prescriptions and limitations: 11.0%). The complete descriptive statistics of the sample are reported in Table 1.

### 3.2. Sickness Events and Relative Absenteeism Days—Description

Over the period 1 January–31 August 2025, overall, the sample produced 745,916 person-days and 509,341 person-workdays. Given that 3016 events and 6630 sickness days were registered, the resulting sickness event rate was 0.54 per 100 person-workdays and the sickness day rate was 0.88 per 100 person-days. The highest event rate was observed among the technical/administrative staff (0.92), whereas the lowest was recorded among the physicians (0.22). As for the absence day rate, the highest was observed in the HSC assistant category (1.41) and the lowest among the physicians (0.31). The complete absenteeism metrics calculated are reported in Table 2.

**Figure 1:**
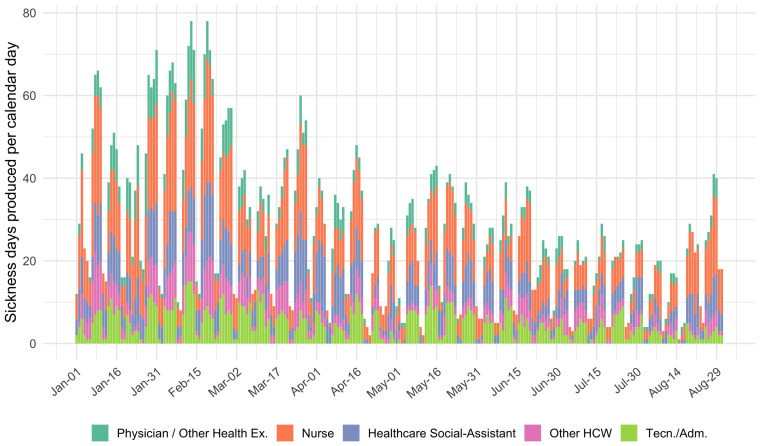
Sickness days produced by the sample of workers over the considered period.

The total sickness days produced by the sample followed a decreasing trend along the considered period. As expected, Saturdays and Sundays, together with national holidays, returned significantly lower values compared to working days, producing a weekly cyclical pattern, as shown in Figure 1. Excluding these days, the absenteeism had its zenith on 13 February (78 absent workers), while the nadir was on 11 August (10 absent workers). The average proportions of absenteeism by job typology were: 41.93% nurses, 23.63% HSC assistants, 14.00% technical/administrative staff, 11.18% other HCWs and 9.26% physicians, partially reflecting the proportion of each job category in the whole sample.

### 3.3. Sickness Events—Model Fit

**Figure 2:**
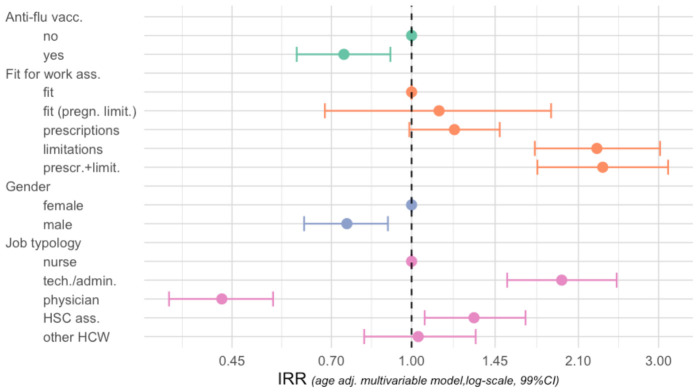
Negative binomial regression coefficients for sickness events expressed as incidence rate ratios (IRRs).

Considering the risk of a sickness event due to over-dispersion of the outcome variable, we employed a negative binomial rather than a Poisson model. Both the uni-variable and the multi-variable coefficients (IRRs) of the sickness event analysis are reported in the Supplementary Materials (Table S2). The multi-variable model fit yielded an IRR of 0.74 (99% CI: 0.60–0.91) for the anti-influenza vaccination, 0.75 (99% CI: 0.62–0.90) for male gender, 2.28 (99% CI: 1.73–3.02) when limitations were applied, and 2.34 (99% CI: 1.75–3.13) for both limitations and prescriptions. With respect to job typology, compared to nurses, the technical/administrative staff carried a higher risk of a sickness short leave (IRR of 1.95; 99% CI 1.53–2.49), as well as compared to the HSC assistants (IRR of 1.32; 99% CI 1.06–1.66). The physician category reported a significant lower risk of event (IRR of 0.43; 99% CI 0.34–0.54). Finally, compared to the “fit for work” individuals, those with limitations presented a more than double risk (IRR of 2.28; 99% CI 1.73–3.02), even when concomitant prescriptions were considered (IRR of 2.34; 99% CI 1.75–3.13). Figure 2 shows the forest plot of the IRRs (along with their 99% CIs) of the multi-variable model fit.

### 3.4. Sickness Days—Model Fit

As for the event variable, due to over-dispersion, a negative binomial model was selected for the sickness day outcome measure. The reference combination of categorical variables was a non-vaccinated female nurse who was assessed as “fit for work” (by protocol, the most frequent combination). The anti-influenza vaccination appeared to be protective both via uni-variable (IRR of 0.53; 99% CI 0.42–0.68) and multi-variable analyses (IRR of 0.74; 99% CI 0.58–0.93). The same was found for male gender, with uni-variable and multi-variable IRRs of 0.66 (99% CI 0.53–0.82) and 0.77 (99% CI 0.63–0.95), respectively. The age coefficient was also significant both in uni-variable (IRR of 1.01; 99% CI 1.00–1.02; *p* = 0.004) and multi-variable analyses (IRR of 0.99; 99% CI 0.98–1.00; *p* = 0.006). The pregnancy limitations were not significant, while the prescriptions were significant regarding the uni-variable (IRR of 1.30; 99% CI 1.02–1.67) but not the multi-variable fit (IRR of 1.22; 99% CI 0.96–1.54). The limitations given regarding the fitness for work assessment increased the risk of sickness days (multi-variable IRR of 2.14; 99% CI 1.51–3.05), as well as when given together with prescriptions (multi-variable IRR of 1.76; 99% CI 1.21–2.55). Considering job typology, compared to nurses, physicians took fewer sickness leaves (multi-variable IRR of 0.36; 99% CI 0.28–0.46); on the contrary, both HSC assistants (multi-variable IRR of 1.43; 99% CI 1.09–1.89) and technical/administrative staff (multi-variable IRR of 1.56; 99% CI 1.15–2.12) reported increased risk. No differences were found in the other professional categories compared to nurses. The complete coefficients of the model fit are reported in Table 3.

## 4. Discussion

The aim of our study was to describe and analyze the sickness absenteeism derived from a short event (5 days maximum) to exclude, at least partially, the effect of severe chronic conditions [25]. The last annual report of the Organization for Economic Co-operation and Development (OECD) [34] about health personnel shows that, across its member countries, nurse is the most represented hospital job typology (36%), followed by physician (14%); healthcare assistants (a category that is equivalent to our HSC assistants) account for circa 20% of the hospital workforce, while nonclinical staff represent roughly a quarter of it. Our sample slightly differs from these numbers, probably because our setting is academic. Given that nurses are, as expected, roughly one third of the HCWs but the number of physicians is inflated by medical residents, the other job proportions are reduced accordingly. Therefore, we reasonably think that our setting is typical for tertiary university hospitals, so our results might be generalizable only to this healthcare facility typology. We found no scientific studies reporting a distribution of workers according to fitness for work assessment results. However, the Piedmont region, which is where our hospital setting is located, published a surveillance report for the year 2021 that claims an overall proportion of 78.2% of workers with a full “fit for work” result (i.e., no prescription or limitation applied), although it aggregates all economic sectors of activity. Another report by the Center on Health and Social Care Management (CeRGAS) of Bocconi University [35] focuses specifically on the healthcare sector: in this work, including more than 130,000 Italian HCWs (circa 25% of the national health system workforce), the prevalence of workers with some limitations accounts for 11.8% of the sample. Considering the job typology, according to this report, some limitations have been applied to 24.4% of HSC workers, 15.1% of nurses, 4.8% of physicians, 8% of other HCWs and between 4.8% and 13.4% for technical/administrative staff. The distribution of this variable in our sample was in line with these numbers, so we reasonably considered that it would not affect the generalizability of our conclusions.

With regard to staff vaccination, the ministerial surveillance PASSI project [36] reported, for the year 2018, the following proportions for anti-influenza vaccination coverage in healthcare facilities: 30.5% for medical doctors (MDs), 9.6% for non-medical health personnel, and 7.5% for non-health personnel. Except for the category of physicians, which appeared quite comparable, we obtained higher proportions of coverage in the non-medical healthcare staff job categories. This result is in line with evidence in the literature that affirms that the level of adhesion to the anti-influenza vaccination campaign has not returned to pre-COVID-19 pandemic levels yet [37]. Notably, our findings and the surveillance data indicate that we are still far from the target of 75% set by the WHO [16] for anti-influenza vaccination of HCWs, even when accounting for a probable minor underestimation (in fact, we did not register as vaccinated those who have been vaccinated outside the hospital campaign, e.g., at their general practitioner office). This data is of particular concern given the literature evidence about the effectiveness of the campaigns: apart from the mentioned systematic review [17], which found for vaccinated workers a standardized mean difference of −0.18 lost working day per person, another large ecological UK NHS study calculated that a 10% increase in vaccination rate is linearly associated with a 10% fall in sickness absence rate [38].

Our outcome metrics of sickness absenteeism are in accordance with the literature since most of the studies report a percentage value of low-to-mid single digits [39]. However, we noticed slightly lower rates, which, in our opinion, may be due to the fact that we considered only the first 8 months of the year, whereas the literature rates are usually calculated annually. It is known, as a matter of fact, that the last months of the year are characterized by disproportionately higher absence rates [40] due to higher circulation of respiratory viruses. We did not include the last months of the year because our results might have been biased by the new upcoming vaccination campaign. In fact, this would have included individuals in our sample who received the vaccination twice along with others who either were not vaccinated or received just a single dose. We therefore reasonably think that our results carry a slight underestimation of the crude absence rates and recommend caution when directly comparing these rates to others, especially if the compared period includes the autumn season. However, we have no reason to think that any covariate included in the analysis disproportionately affects absence rates because no seasonal effect of any of them has been found in the literature.

The results of the event analysis (both the comparison of crude rates and the model fit coefficients) closely resemble those concerning sickness days, as can be inferred from the fact that we considered only short events. In fact, during the overall period, our workers generated a mean of 0.94 short events (SD: 1.92) per capita and a mean of 1.04 days (SD: 1.46) per short event. Table S3 and Figures S1 and S2 in the Supplementary Materials report all the sickness days and events (both long and short) generated by the cohort during the considered period, showing that, while long events represent a small proportion of the total sickness events, they account for the majority of days. This probably confirms that, the shorter the duration of the events, the higher the correlation between events and sickness days may become; therefore, when considering absenteeism derived from short events, it should be sufficient to analyze the sickness days only. The double aspect analysis (both events and days) may be meaningful only when all the absence days and events are included in the analysis.

In our study, we found that anti-influenza vaccination appeared to be protective against sickness absence, as already confirmed in the literature: a recent systematic review has calculated, for vaccinated workers, an absenteeism risk ratio of 0.63 (95% CI: 0.46–0.86) [17]. Another study estimated a vaccine efficacy against influenza-related absenteeism of 44.8% (95% CI: 22.8–60.6%) in the healthcare sector [41]. Finally, a relevant review reported that influenza-vaccinated HCWs had less excess absenteeism compared with unvaccinated ones (1.74 versus 2.71 days/employee) and confirmed the effectiveness of vaccination against COVID-19 [42]. Based on the literature data and our analysis, we conclude that our results reaffirm the recommendations of the WHO and the Italian Ministry of Health [43], stressing the importance of any measure supporting uptake. We may also conclude that vaccination is useful even accounting for age and, most of all, job typology, regardless of the biological risk incurred. If confirmed, this would mean no particular prioritization strategies based on job typology or age should be applied to increase effectiveness.

We found that female gender is associated with more absence sickness days. As reported by a systematic review, this finding is already pointed out by several studies in the literature, not only in the healthcare sector [44,45]. Possible explanations for this phenomenon are major expression of health needs by females, pregnancy and its implications, possible different diagnostic behavior of physicians depending on gender, overload due to work and personal family needs, and increased susceptibility to work stressors. Even if we found a significant coefficient for the age covariate, given the magnitude of the effect (0.99), we consider its effect negligible both from organizational and clinical points of view. Notably, the literature evidence about an age effect on sickness leaves reports disproportionate growth when the age of the workers is over 60 [33]. Overall, our sample reported a mean age of 46, which is significantly lower and, therefore, probably insufficient to appreciate any meaningful effect.

Concerning job typology, no difference was found between nurses and other HCWs; we therefore conclude that this is an unnecessary distinction in the analysis of absenteeism unless a contractual or other administrative aspect is under investigation since the internal risk assessment document already identifies the same occupational health risks for both. Based on our findings, we agree with the literature about the fact that physicians tend to produce fewer sickness absence days, sometimes experiencing the opposite “presenteeism” phenomenon, a recognized risk both for the mental health of the professional and the facility, which may encounter organizational problems [46,47]. Even though precise causative mechanisms are partially unknown, possible explanations are related to susceptible personality, job satisfaction [48], work culture, remorse for abandoning patients or difficulty in playing the role of a patient [49,50]. Also, we reasonably think that the possibility of auto-prescription or self-medication could at least in part explain this finding. Further research investigating this aspect should necessarily employ qualitative or mixed-method techniques. We found that HSC assistants carry more risk of sickness absence, even accounting for possible limitations. We retrieved no study for a direct comparison, but we reasonably think that the same determinants regarding physicians apply to this job category but in the opposite direction: it is known, in fact, that this kind of job, with limited autonomy and high manual workload shifts, is associated with burnout [51] and, in turn, sickness absenteeism. Notably, we noticed a high risk for the technical/administrative category, which is counterintuitive given that the only health risk identified by the mandatory assessment is prolonged computer use. We therefore think that either job satisfaction is disproportionately low in this category (actually, the majority of the workers assessed as “unfit for work” at their job are re-assigned to this one, losing contact with patient care; also, high bureaucracy, typical of administrative work, is known to be associated with low job satisfaction [52]) or health risk may be underestimated for this job typology. However, we did not have the opportunity to include the precise cause of the sickness event in the analysis by law (the employer is forbidden to know details about the health status of the employee to fight work discrimination); therefore, qualitative research investigating the perception of single individuals should be conducted for this purpose, probably with a direct survey (after careful assessment of participation bias).

The most original aspect of our research is the inclusion of the fitness for work assessment as a covariate, also adjusting for gender, age, vaccination and job typology. We did not expect any difference in the absence rates because, when any prescription or limitation is applied, certain job tasks are either not performed or are performed using additional equipment, preserving, at least in theory, the already compromised health of the worker. For the 80 pregnant workers, in fact, no statistically significant difference in risk was observed despite quite heavy job limitations (no night shifts, no biological hazard exposure, no prolonged standing tasks, etc.). However, the study was underpowered for detecting any effect in such a small subgroup sample size. Also, the presence of any prescription did not result in a significant risk increase. On the contrary, an applied limitation significantly increased the risk of short sickness absence by more than two-fold (a little less when applied in combination with a prescription). We speculate the following explanations for this finding: first, limitations may not be followed on a daily regular basis as expected. Even though some workers or executives could sometimes neglect these mandatory restrictions, we reasonably think that the public vigilance authority offices would have intervened in the case of massive violations that more than double the health risk of the worker. Another reason could be that a worker with a limitation, even though job-specific, could be considered a “fragile”, or, better, more vulnerable individual in a broader nosological sense of the term, and not only with regard to the health conditions that determined the limitation itself. For example, a worker with a heavy lifting limitation may also have an increased susceptibility to aerial infections, resulting in higher sickness days. The underlying biological mechanisms, however, will remain unknown until precise hypotheses are formulated and further studies (preferably with higher levels of evidence) specifically designed for this purpose are conducted.

Finally, we must acknowledge that limitations are only active for the working hours of the individual (circa 38 h/week), but he/she may be at risk even outside this timespan because of his/her private life activities. For instance, a chemical compound removed from the workplace of a susceptible individual can cause harm to the same subject if the exposure persists at home, resulting in higher sickness absence days. Thus, the limited worker might be considered more “fragile” (or vulnerable), even beyond temporal (as well as nosological) limits.

### Strengths and Limitations

The strength of the study, as well as its original aspect, is the incorporation of the fitness for work assessment into the analysis of absence events. To our knowledge, no other study has explored this relationship. This is of paramount importance when allocating human resources because, in hospitals, there are activities whose interruption is not possible without causing harm to the patient or increasing clinical risk. Knowing in advance any pattern of absence by the typology of the worker allows the organizational offices to minimize any problem in delivering proper care at the right time. Another strength of the study is its robust data analysis: the forecasted large sample of workers who were eligible for inclusion allowed the choice of an alpha set at 0.01 and the construction of 99% CIs.

The main limitation of our study is the exclusion of the causes of the sickness events from the analysis because of the inability to access the data due to legal restrictions. Even though the literature reports that the majority of sickness leaves are due to ILIs, followed by musculoskeletal problems [6], we were not able to verify in practice the real proportions. This is important when considering the impact of vaccinations because alternative hypotheses should be generated if the proportion of sickness absence days due to infectious respiratory diseases turns out to be sufficiently low. Another limitation arises because, when a holiday or weekend fell in between two sickness days, we considered the sickness event uninterrupted and counted it as a single event. Accounting for this choice, the sickness events in our sample had a median duration of 2 (IQR 1–3). Therefore, the majority of events did not need this correction, and we reasonably think that this methodological choice could not have had a significant impact on our results or conclusions. The study timespan did not include the last four months of the year because we chose not to conduct the analysis on a mixed-dynamic population. This choice is not common in the literature and limits the comparability of our crude rates to other studies, but we reasonably think that this factor does not disproportionately affect any covariate and therefore represent a bias in our conclusions. Also, we did not incorporate any job-related variables, such as mental status and/or job satisfaction, which are known to play a significant role in absenteeism [7,53]. Should these variables also be strongly linked to particular jobs, vaccination propensity or fitness for work assessment, our results may be partially biased. Moreover, we reaffirm that we focused only on short sickness events, so our conclusions should be read with caution when comparing them to the overall absence rates because the determinants of sickness leave could be different between short and long events.

Finally, a major limitation of our work is that we did not include the ward typology (or the department) of the worker in the analysis. It is known that some areas of the hospital (e.g., the emergency department) carry a higher risk of sickness (e.g., ILIs) due to prolonged contact with patients [20]. Should this factor be strongly associated with one or more of our grouping variables, our results may be significantly biased. However, some job categories may be more likely to be employed in a specific department so that strong imbalances may be mitigated, and information about ward typology may be, at least partially, included in the job typology variable. However, our suggestion is that future research about sickness absenteeism in the healthcare sector should explicitly take this variable into account.

## 5. Conclusions

Our study showed that, in a large tertiary university hospital, an anti-influenza vaccination campaign seems to effectively protect workers from sickness absences, even accounting for gender, age and regardless of job typology and fitness for work assessment. Furthermore, the health risk of the technical/administrative job category might be underestimated, and further research is recommended. Finally, our results suggest that the worker with applied limitations might be “fragile”, or vulnerable, in a broader sense of the term, both from nosological and temporal points of view. Further research with a higher level of evidence is needed to verify and confirm these findings.

## Figures and Tables

**Table 1:** Description of the study sample of hospital workers.

Label	Modality	Overall	Nurse	Technical/Administrative	Physician	Health and Social Care Assistant	Other HCW
n		3218	1073 (33.3)	357 (11.0)	976 (30.3)	454 (14.1)	358 (11.1)
Gender	Female	2317 (72.0)	848 (79.0)	248 (69.5)	570 (58.4)	380 (83.7)	271 (75.7)
	Male	901 (28.0)	225 (21.0)	109 (30.5)	406 (41.6)	74 (16.3)	87 (24.3)
Age (yr) —mean (SD)		46.06 (11.85)	45.75 (11.36)	54.05 (9.10)	41.84 (11.62)	50.98 (9.83)	44.27 (12.57)
Flu vaccination	No	2534 (78.7)	911 (84.9)	294 (82.4)	634 (65.0)	403 (88.8)	292 (81.6)
	Yes	684 (21.3)	162 (15.1)	63 (17.6)	342 (35.0)	51 (11.2)	66 (18.4)
Fit for work assessment	Fit for work	2131 (66.2)	655 (61.0)	257 (72.0)	764 (78.3)	228 (50.2)	227 (63.4)
	Fit with pregnancy limitations	80 (2.5)	20 (1.9)	3 (0.8)	38 (3.9)	3 (0.7)	16 (4.5)
	Fit with limitations	222 (6.9)	88 (8.2)	22 (6.2)	29 (3.0)	52 (11.5)	31 (8.7)
	Fit with prescriptions	584 (18.1)	219 (20.4)	50 (14.0)	134 (13.7)	121 (26.7)	60 (16.8)
	Fit with prescriptions and limitations	201 (6.2)	91 (8.5)	25 (7.0)	11 (1.1)	50 (11.0)	24 (6.7)

**Table 2:** Crude and job-specific sickness absenteeism metrics of the sample.

	Overall	Nurse	Technical/Administrative	Physician	HSC Assistant	Other HCW
Total person-workdays	754,916	253,569	84,116	228,251	105,570	83,410
Total person-workdays	509,096	170,968	56,533	154,554	70,879	56,162
Total sickness events (≤5 days)	2771	1028	516	336	543	348
Total sickness days (≤5 days)	6630	2530	1071	713	1488	828
Sickness event rate (per 100 person-workdays)	0.54	0.60	0.92	0.22	0.77	0.62
Sickness day rate (per 100 person-days)	0.88	1.00	1.27	0.31	1.41	0.99

**Table 3:** Incidence rate ratios (IRRs) from a negative binomial model for sickness days due to short events (≤5 days). Statistically significant results are marked with a *.

Variable	Modality	IRR Uni-Variable (99% CI)	IRR Multi-Variable (99% CI)
Anti-flu vaccination	No (Ref.)	-	-
	Yes	0.53 (0.42–0.68) *	0.74 (0.58–0.93) *
Gender	F (Ref.)	-	-
	M	0.66 (0.53–0.82) *	0.77 (0.63–0.95) *
Age		1.01 (1.00–1.02) *	0.99 (0.98–1.00) *
Fitness for work ass.	Fit for work (Ref.)	-	-
	Fit with pregnancy limitations	1.09 (0.60–1.99)	1.22 (0.68–2.19)
	Fit with prescriptions	1.30 (1.02–1.67) *	1.22 (0.96–1.54)
	Fit with limitations	2.27 (1.58–3.27) *	2.14 (1.51–3.05) *
	Fit with presc. and limitations	2.13 (1.46–3.12) *	1.76 (1.21–2.55) *
Job typology	Nurse (Ref.)	-	-
	Technical/administrative	1.33 (0.99–1.80)	1.56 (1.15–2.12) *
	Physician (or equiv.)	0.31 (0.25–0.40) *	0.36 (0.28–0.46) *
	Healthcare social assistant	1.43 (1.08–1.88) *	1.43 (1.09–1.89) *
	Other healthcare worker	1.01 (0.75–1.37)	0.99 (0.73–1.34)

## Data Availability

Data is available upon motivated request.

## Supplementary Materials

The following supporting information can be downloaded at: https://www.mdpi.com/article/10.3390/epidemiologia7040096/s1, Table S1: STROBE statement—checklist of items that should be included in reports of cohort studies; Table S2: Negative binomial model coefficients (IRRs) for the sickness events; Table S3: Complete absenteeism metrics of the cohort, including short and long events; Figure S1: Total sickness events produced by the cohort during the period (1 January–31 August 2025); Figure S2: Total sickness days produced by the cohort during the period (1 January–31 August 2025). Figure S3: Facet Wrap of Figure 1 Sickness days produced by the different groups of worker per calendar day.

## Abbreviations

The following abbreviations are used in this manuscript:

GDP	Gross Domestic Product
NIOSH	National Institute for Occupational Safety and Health
OR	Odds Ratio
CI	Confidence Interval
ILI	Influenza-Like Illness
HSC	Health and Social Care
HCW	Health Care Worker
IRR	Incidence Rate Ratio
