# Supplementary material for: Short Sickness Absenteeism Rate in a Large Tertiary Hospital: The Role of Fitness for Work and Influenza Vaccination
Source: Epidemiologia (Basel). 2026 Jul 9;7(4):96. doi: 10.3390/epidemiologia7040096 (PMC13397925; doi:10.3390/epidemiologia7040096)
Supplement: Supplementary file 1 [file epidemiologia-07-00096-s001.zip › epidemiologia-4300781-supplementary.pdf]

SUPPLEMENTARY MATERIALS

Short Sickness Absenteeism Rate in a Large Tertiary Hospital and the Role of Fitness for Work and Influenza Vaccination : an observational cohort study

*Alberto Lontano, Luca Inguaggiato, Daniele Ceriotti, Riccardo Rescinito ,Enrico Oddone, Stefano M Candura, and Matteo Ratti**

Table S1 - STROBE Statement—Checklist of items that should be included in reports of cohort studies

	Item No	Recommendation	Section / Page
**Title and abstract**	1	(*a*) Indicate the study's design with a commonly used term in the title or the abstract	Page 1 – see abstract, section background: an observational cohort study
		(*b*) Provide in the abstract an informative and balanced summary of what was done and what was found	Page 1 – see Abstract
**Introduction**			
Background/rationale	2	Explain the scientific background and rationale for the investigation being reported	Page 2 – section 1 Introduction
Objectives	3	State specific objectives, including any prespecified hypotheses	Page 3 – section 1 Introduction
**Methods**			
Study design	4	Present key elements of study design early in the paper	Page 3 – section 2.1 Study aim and Design
Setting	5	Describe the setting, locations, and relevant dates, including periods of recruitment, exposure, follow-up, and data collection	Page 3- section 2.2 (Study Popoulation) and page 4 – section 2.4 (Data collection and management)
Participants	6	(*a*) Give the eligibility criteria, and the sources and methods of selection of participants. Describe methods of follow-up	Page 3 – section 2.2 (Study Popoulation)
		(*b*) For matched studies, give matching criteria and number of exposed and unexposed	Not applicable
Variables	7	Clearly define all outcomes, exposures, predictors, potential confounders, and effect modifiers. Give diagnostic criteria, if applicable	Page 4 – section 2.3 (Study outcomes) and page 5 – section 2.6 (Potential bias and confounders)
Data sources/ measurement	8*	For each variable of interest, give sources of data and details of methods of assessment (measurement). Describe comparability of assessment methods if there is more than one group	Page – section 2.4 – Data collection and management
Bias	9	Describe any efforts to address potential sources of bias	Page – section 2.6 Potential bias and confounders

Study size	10	Explain how the study size was arrived at	Page 5 – section 2.5 (Data Analysis)
Quantitative variables	11	Explain how quantitative variables were handled in the analyses. If applicable, describe which groupings were chosen and why	Page 4/5 – section 2.5 (Data Analysis)
Statistical methods	12	(*a*) Describe all statistical methods, including those used to control for confounding	Page 4/5 – section 2.5 (Data Analysis)
		(*b*) Describe any methods used to examine subgroups and interactions	Page 4/5 – section 2.5 (Data Analysis)
		(*c*) Explain how missing data were addressed	Not applicable
		(*d*) If applicable, explain how loss to follow-up was addressed	Not applicable
		(*e*) Describe any sensitivity analyses	Not applicable
**Results**			
Participants	13*	(a) Report numbers of individuals at each stage of study—eg numbers potentially eligible, examined for eligibility, confirmed eligible, included in the study, completing follow-up, and analysed	Page 5 – section 3.1 (Sample description)
		(b) Give reasons for non-participation at each stage	Not applicable
		(c) Consider use of a flow diagram	Not applicable
Descriptive data	14*	(a) Give characteristics of study participants (eg demographic, clinical, social) and information on exposures and potential confounders	Page 5 – section 3.1 and page 6 - Table 1
		(b) Indicate number of participants with missing data for each variable of interest	Not applicable
		(c) Summarise follow-up time (eg, average and total amount)	Page 6 – Table 2
Outcome data	15*	Report numbers of outcome events or summary measures over time	Page 6 – Table 2
Main results	16	(*a*) Give unadjusted estimates and, if applicable, confounder-adjusted estimates and their precision (eg, 95% confidence interval). Make clear which confounders were adjusted for and why they were included	Page 7 – Figure 2 and section 3.3<br><br>Page 9 – Table 3 and section 3.4
		(*b*) Report category boundaries when continuous variables were categorized	Not applicable
		(*c*) If relevant, consider translating estimates of relative risk into absolute risk for a meaningful time period	Not applicable
Other analyses	17	Report other analyses done—eg analyses of subgroups and interactions, and sensitivity analyses	Supplementary materials
**Discussion**			
Key results	18	Summarise key results with reference to study objectives	Pages 9/10
Limitations	19	Discuss limitations of the study, taking into account sources of potential bias or imprecision. Discuss both direction and magnitude of any potential bias	Pages 11/12 – sect. 4.1 (Strengths and limitations)
Interpretation	20	Give a cautious overall interpretation of results considering objectives, limitations, multiplicity of analyses, results from similar studies, and other relevant evidence	Page 11- section 4 Discussion
Generalisability	21	Discuss the generalisability (external validity) of the study results	Pages 9/10 – section 4 Discussion
**Other information**			
Funding	22	Give the source of funding and the role of the funders for the present study and, if applicable, for the original study on which the present article is based	Not applicable

*Give information separately for exposed and unexposed groups.

Note: An Explanation and Elaboration article discusses each checklist item and gives methodological background and published examples of transparent reporting. The STROBE checklist is best used in conjunction with this article (freely available on the Web sites of PLoS Medicine at http://www.plosmedicine.org/, Annals of Internal Medicine at http://www.annals.org/, and Epidemiology at http://www.epidem.com/). Information on the STROBE Initiative is available at http://www.strobe-statement.org.

Table S2 - Negative Binomial Model Coefficients (IRRs) for the sickness events.

Variable	Modality	IRR Uni-variable (99% CI)	IRR Multi-variable (99% CI)
Anti-flu vaccination	No (Ref.)	-	-
	Yes	0.54 (0.44 – 0.66)*	0.74 (0.60 – 0.91)*
Gender	F (Ref.)	-	-
	M	0.64 (0.53 – 0.77)*	0.75 (0.62 – 0.90)*
Age		1.01 (1.00 – 1.01)	0.98 (0.98 - 0.99)*
Fitness to work assessment	Fit for work (Ref.)	-	-
	Fit with pregnancy limitations	1.14 (0.69 – 1.90)	1.13 (0.68 – 1.86)
	Fit with prescriptions	1.27 (1.04 – 1.56)*	1.21 (0.99 – 1.48)
	Fit with limitations	2.39 (1.80 – 3.17)*	2.28 (1.73 – 3.02)*
	Fit with presc. and limitations	2.55 (1.90 – 3.43)*	2.34 (1.75 – 3.13)*
Job Typology	Nurse (Ref.)	-	-
	Technical / Administative	1.57 (1.23 – 2.00)*	1.95 (1.53 – 2.49)*
	Physician or equivalent	0.36 (0.29 – 0.45)*	0.43 (0.34 – 0.54)*
	Healthcare Social Assistant	1.29 (1.03 – 1.63)*	1.32 (1.06 – 1.66)*
	Other Healthcare Worker	1.06 (0.82 – 1.36)	1.03 (0.81 – 1.33)

Table S3 - Complete absenteeism metricsof the cohort including short and long events

Job Typology	Person-days	Person-workdays	Sickness events	Sickness days	Sickness day rate	Sickness event rate
**Techn. /Admin.**	85’790	56’624	607	2’745	3.20	1.07
**Physician**	229’116	154’592	374	1’578	0.69	0.24
**Nurse**	257’208	171’204	1’264	6’169	2.40	0.74
**HSC ass.**	108’784	71’097	761	4’702	4.32	1.07
**Other HCW**	84’759	56’237	423	2’177	2.57	0.75
**Total**	765’657	509’754	3’429	17’371	2.27	0.67

Figure S1 - Total sickness events produced by the cohort during the period (Jan 1st – Aug 31st, 2025)

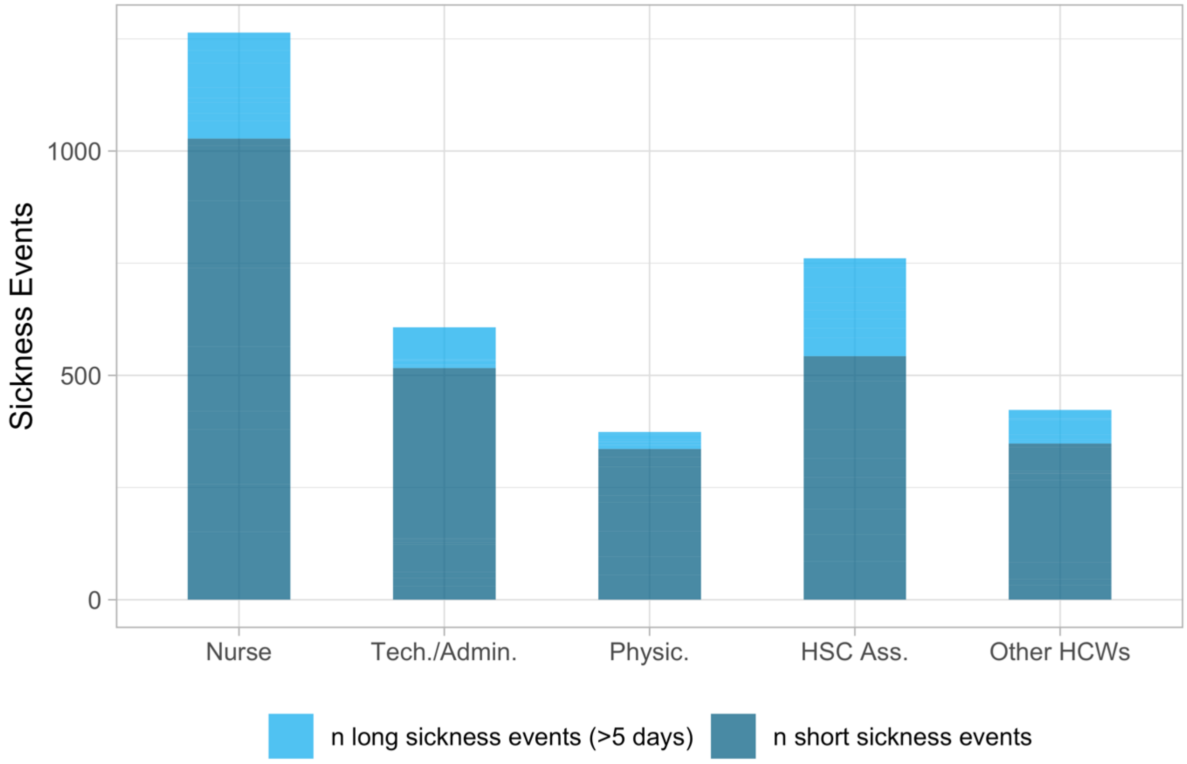

Figure S2 - Total sickness days produced by the cohort during the period (Jan 1st – Aug 31st, 2025)

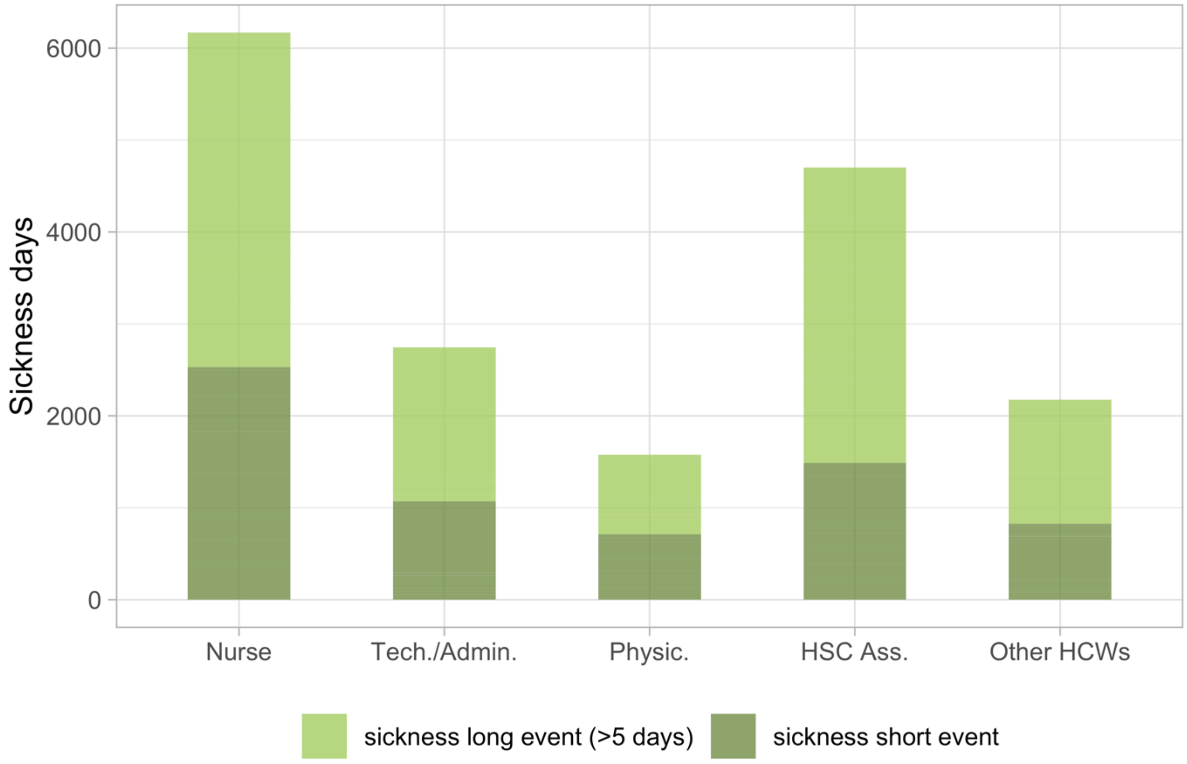

Figure S3 - Facet Wrap of Figure 1. Sickness days produced by the different groups of worker per calendar day

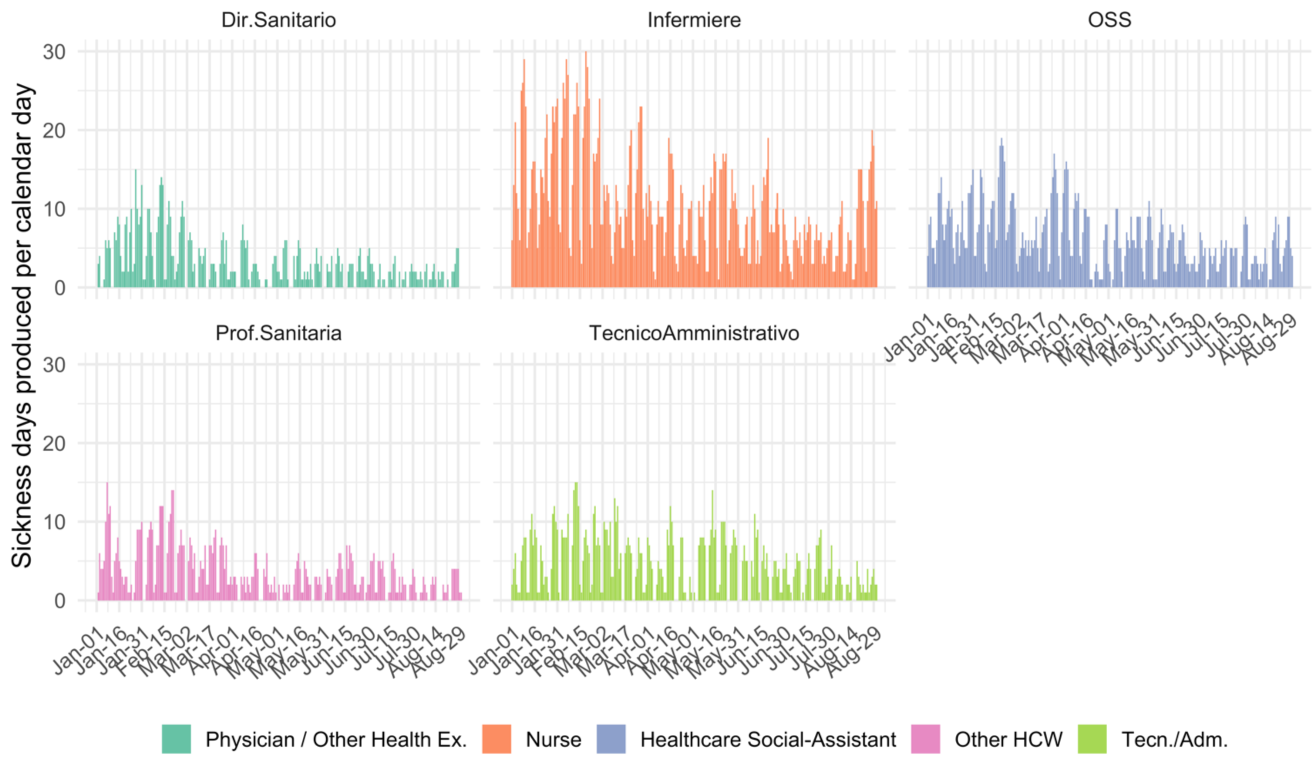

Model code and Rationale

Given that the outcome variable is a numerical count, we employed a Poisson Regression for the preliminary analysis. To check the overdispersion, a preliminary multi-variable model has been tried with the following code:

```
fit <- glm(ggMalattia ~ FLU_YESNO+TIPO_LAV+Giudizio+ETA+GENERE,
        family = poisson(link = "log"), data = db_dip)
disp <- sum(residuals(fit, type = "pearson")^2) / df.residual(fit)
disp

[1] 37.07005
```

Where ggMalattia is the outcome variable (sickness days), FLU_YES_NO is the flag of being vaccinated against seasonal influenza, TIPO_LAV is the job typology, Giudizio is the result of the fit for work assessment visit, ETA is Age, and GENERE is Gender.

The output value is way more than 1, suggesting a strong overdispersion of the outcome variable. Therefore, for every variable in a loop labeled as i we applied a Negative Binomial model as follows:

```
my_formula <- as.formula(paste("ggMalattia ~ ",i, " +offset(log(ggProdotte))"))
mod_nb <- glm.nb(my_formula, data = db_dip)
disp_nb <- sum(residuals(mod_nb, type = "pearson")^2) / df.residual(mod_nb)
   print(paste("Dispersione:",round(disp_nb,2)))
```

Results of the model with outcome: **number of events:**

```
Call:
glm.nb(formula = nEventi_max_5 ~ FLU_YESNO + TIPO_LAV + ETA +
    Giudizio + GENERE + offset(log(ggLavorative_5)), data = db_dip,
    init.theta = 0.7097331184, link = log)

Coefficients:
                                                           Estimate Std. Error z value Pr(>|z|)
(Intercept)                                               -4.475565   0.135800 -32.957  < 2e-16
FLU_YESNOYES                                              -0.307506   0.081117  -3.791  0.00015
TIPO_LAVTecnicoAmministrativo                              0.668305   0.095282   7.014 2.32e-12
TIPO_LAVDir.Sanitario                                     -0.849232   0.086928  -9.769  < 2e-16
TIPO_LAVOSS                                                0.280982   0.087465   3.213  0.00132
TIPO_LAVProf.Sanitaria                                     0.034021   0.097395   0.349  0.72685
ETA                                                       -0.016614   0.002855  -5.819 5.93e-09
GiudizioIDONEO CON LE LIMITAZIONI PREVISTE DAL D.Lgs. 26.3.2001 N. 151  0.117917   0.194709   0.606  0.54478
GiudizioIDONEO CON LIMITAZIONI                             0.826301   0.108369   7.625 2.44e-14
GiudizioIDONEO CON PRESCRIZIONI                            0.193283   0.077970   2.479  0.01318
GiudizioIDONEO CON PRESCRIZIONI E LIMITAZIONI              0.851765   0.112568   7.567 3.83e-14
GENEREM                                                   -0.289850   0.071538  -4.052 5.09e-05

(Intercept)                                               ***
FLU_YESNOYES                                              ***
TIPO_LAVTecnicoAmministrativo                             ***
TIPO_LAVDir.Sanitario                                     ***
TIPO_LAVOSS                                               **
TIPO_LAVProf.Sanitaria
ETA                                                       ***
GiudizioIDONEO CON LE LIMITAZIONI PREVISTE DAL D.Lgs. 26.3.2001 N. 151
GiudizioIDONEO CON LIMITAZIONI                            ***
GiudizioIDONEO CON PRESCRIZIONI                           *
GiudizioIDONEO CON PRESCRIZIONI E LIMITAZIONI             ***
GENEREM                                                   ***
---
Signif. codes:  0 ‘***’ 0.001 ‘**’ 0.01 ‘*’ 0.05 ‘.’ 0.1 ‘ ’ 1

(Dispersion parameter for Negative Binomial(0.7097) family taken to be 1)

    Null deviance: 3198.2  on 3217  degrees of freedom
Residual deviance: 2754.1  on 3206  degrees of freedom
AIC: 7834.2

Number of Fisher Scoring iterations: 1


              Theta:  0.7097
          Std. Err.:  0.0412

 2 x log-likelihood:  -7808.1900
[1] "Dispersione: 1.3"
                                                           IRR CI_lower CI_upper
(Intercept)                                               0.01     0.01     0.02
FLU_YESNOYES                                              0.74     0.60     0.91
TIPO_LAVTecnicoAmministrativo                             1.95     1.53     2.49
TIPO_LAVDir.Sanitario                                     0.43     0.34     0.54
TIPO_LAVOSS                                               1.32     1.06     1.66
TIPO_LAVProf.Sanitaria                                    1.03     0.81     1.33
ETA                                                       0.98     0.98     0.99
GiudizioIDONEO CON LE LIMITAZIONI PREVISTE DAL D.Lgs. 26.3.2001 N. 151 1.13     0.68     1.86
GiudizioIDONEO CON LIMITAZIONI                            2.28     1.73     3.02
GiudizioIDONEO CON PRESCRIZIONI                           1.21     0.99     1.48
GiudizioIDONEO CON PRESCRIZIONI E LIMITAZIONI             2.34     1.75     3.13
GENEREM                                                   0.75     0.62     0.90
```

Calculated dispersion: 1.3

Results of the model with outcome variable: **sickness days**

```
Call:
glm.nb(formula = ggMalattia_max_5 ~ FLU_YESNO + TIPO_LAV + ETA +
    Giudizio + GENERE + offset(log(ggPeriodo_5)), data = db_dip,
    init.theta = 0.3178930665, link = log)

Coefficients:
                                                                 Estimate Std. Error z value Pr(>|z|)
(Intercept)                                                     -4.276546   0.159327 -26.841  < 2e-16
FLU_YESNOYES                                                    -0.307510   0.090178  -3.410 0.000650
TIPO_LAVTecnicoAmministrativo                                    0.443995   0.118609   3.743 0.000182
TIPO_LAVDir.Sanitario                                           -1.011068   0.094945 -10.649  < 2e-16
TIPO_LAVOSS                                                      0.360303   0.106578   3.381 0.000723
TIPO_LAVProf.Sanitaria                                          -0.008156   0.116709  -0.070 0.944289
ETA                                                             -0.009010   0.003298  -2.732 0.006294
GiudizioIDONEO CON LE LIMITAZIONI PREVISTE DAL D.Lgs. 26.3.2001 N. 151  0.199515   0.226576   0.881 0.378553
GiudizioIDONEO CON LIMITAZIONI                                   0.762687   0.136248   5.598 2.17e-08
GiudizioIDONEO CON PRESCRIZIONI                                  0.198514   0.091618   2.167 0.030253
GiudizioIDONEO CON PRESCRIZIONI E LIMITAZIONI                    0.564529   0.143839   3.925 8.68e-05
GENEREM                                                         -0.256897   0.081059  -3.169 0.001528

(Intercept)                                                     ***
FLU_YESNOYES                                                    ***
TIPO_LAVTecnicoAmministrativo                                   ***
TIPO_LAVDir.Sanitario                                           ***
TIPO_LAVOSS                                                     ***
TIPO_LAVProf.Sanitaria
ETA                                                             **
GiudizioIDONEO CON LE LIMITAZIONI PREVISTE DAL D.Lgs. 26.3.2001 N. 151
GiudizioIDONEO CON LIMITAZIONI                                  ***
GiudizioIDONEO CON PRESCRIZIONI                                 *
GiudizioIDONEO CON PRESCRIZIONI E LIMITAZIONI                   ***
GENEREM                                                         **
---
Signif. codes:  0 ‘***’ 0.001 ‘**’ 0.01 ‘*’ 0.05 ‘.’ 0.1 ‘ ’ 1

(Dispersion parameter for Negative Binomial(0.3179) family taken to be 1)

    Null deviance: 3050.7  on 3217  degrees of freedom
Residual deviance: 2723.0  on 3206  degrees of freedom
AIC: 10996

Number of Fisher Scoring iterations: 1


              Theta:  0.3179
          Std. Err.:  0.0131

 2 x log-likelihood:  -10969.6080
[1] "Dispersione: 0.91"
                                                                IRR CI_lower CI_upper
(Intercept)                                                     0.01     0.01     0.02
FLU_YESNOYES                                                    0.74     0.58     0.93
TIPO_LAVTecnicoAmministrativo                                   1.56     1.15     2.12
TIPO_LAVDir.Sanitario                                           0.36     0.28     0.46
TIPO_LAVOSS                                                     1.43     1.09     1.89
TIPO_LAVProf.Sanitaria                                          0.99     0.73     1.34
ETA                                                             0.99     0.98     1.00
GiudizioIDONEO CON LE LIMITAZIONI PREVISTE DAL D.Lgs. 26.3.2001 N. 151 1.22     0.68     2.19
GiudizioIDONEO CON LIMITAZIONI                                  2.14     1.51     3.05
GiudizioIDONEO CON PRESCRIZIONI                                 1.22     0.96     1.54
GiudizioIDONEO CON PRESCRIZIONI E LIMITAZIONI                   1.76     1.21     2.55
GENEREM                                                         0.77     0.63     0.95
```

Calculated dispersion: 0.91
